# Optimization of the CRISPR/Cas9 system using *adh1* promoter derivatives in fission yeast

**DOI:** 10.17912/micropub.biology.000757

**Published:** 2023-02-03

**Authors:** Miori Saito, Hidenori Nakaoka, Aki Hayashi, Hiroaki Takaku, Harutake Yamazaki

**Affiliations:** 1 Department of Applied Life Sciences, Niigata University of Pharmacy and Applied Life Sciences, Niigata, Niigata, Japan; 2 Graduate School of Biostudies, Kyoto University, Kyoto, Kyoto, Japan; 3 Division of Chromatin Regulation, National Institute for Basic Biology, Okazaki, Aichi, Japan

## Abstract

The CRSIPR/Cas9 system has been applied to fission yeast, but there remain some rooms for improvement. Here we report that the weaker versions of the *adh1* ^+^ promoter, *adh11* and *adh41* promoters, for the potentially cytotoxic Cas9 achieved highly efficient mutagenesis and gene deletion at the *ade6* ^+^ locus. Employing a drug-selectable marker instead of conventional auxotrophic markers, our new vector system is compatible with a variety of experimental settings including prototrophic/auxotrophic strains and complete/minimal media.

**Figure 1. Efficient mutagenesis and gene deletion using adh11 and adh41 promoters for expression of Cas9:**
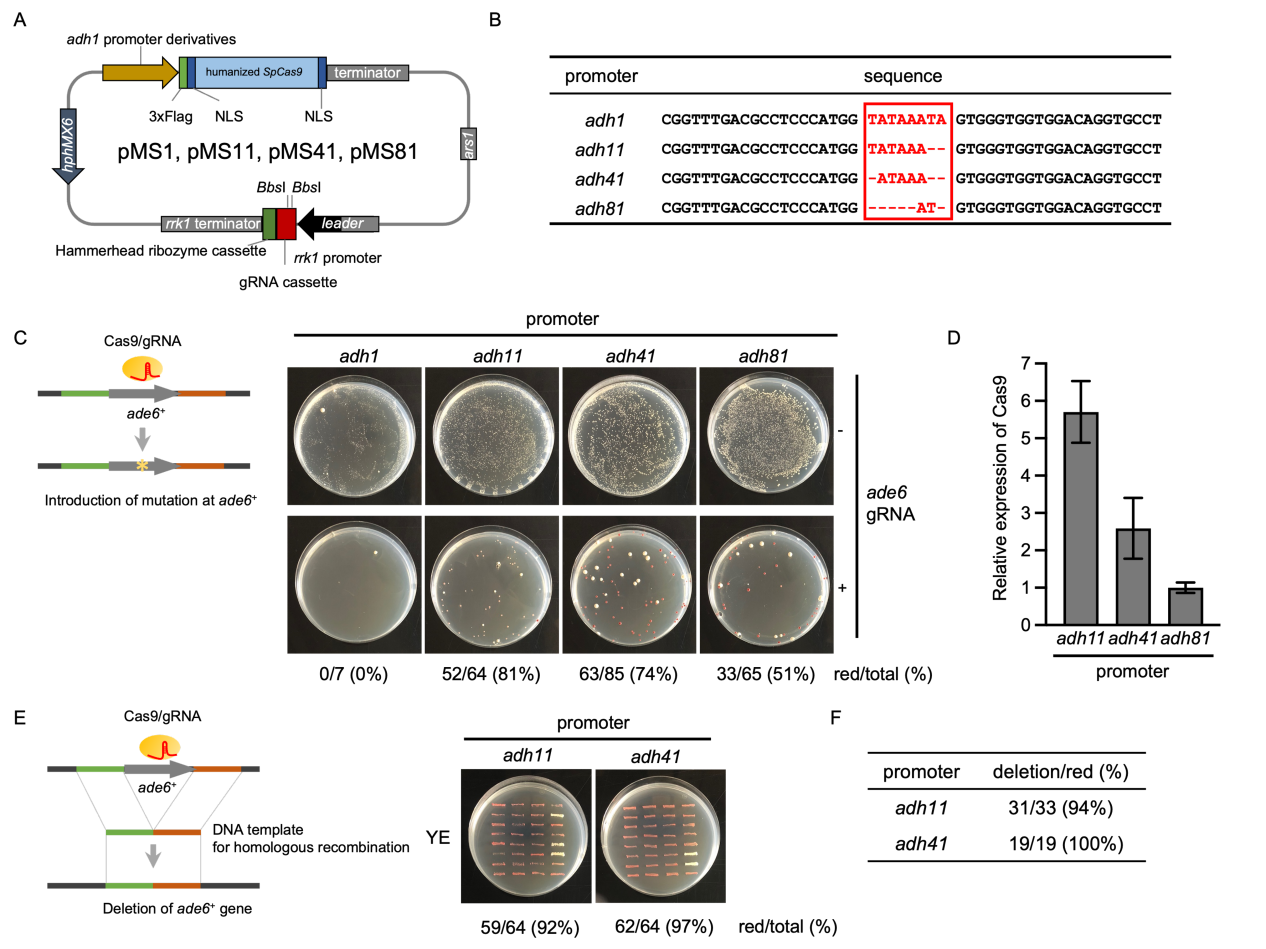
(A) Map of pMS1, pMS11, pMS41, and pMS81. (B) Sequence alignment of the TATA box (in the red box) and its flanking sequences in *adh1* promoter derivatives. Nucleotide sequences about 100 bp upstream from the START codon of *adh1* ^+^ are shown. (C) CRISPR/Cas9-mediated mutagenesis at the *ade6* ^+^ locus. Typical examples of the selective plates after transformation by the empty vectors (top panels) and gRNA expression vectors (bottom panels) are shown. Introduction of the mutations can be easily identified by the characteristic red color of the colonies. (D) Relative expression levels of Cas9 to *act1* ^+^ in cells with pMS11, pMS41, or pMS81. Each bar shows the mean value of three biological replicates, which is further normalized to that of *adh81.* Error bars represent SEM. The lack of data for the *adh1* promoter is due to the extremely poor growth of the cells transformed with pMS1 in YES containing hygromycin B. (E) CRISPR/Cas9-mediated gene deletion at the *ade6* ^+^ locus. Wild-type cells were transformed by pMS11_ *ade6* -gRNA or pMS41_ *ade6* -gRNA with the DNA template for gene deletion by homologous recombination. Randomly picked-up transformants were restreaked on YE plates, and the appearance of the red-colored colonies (i.e., mutation or gene deletion of the *ade6* ^+^ gene) was confirmed. (F) Efficiency of deletion at the *ade6* ^+^ gene. The red-colored cells in (E) were subjected to colony PCR to confirm the successful deletion of the *ade6* ^+^ gene.

## Description

The CRISPR/Cas9 system has been adapted to *Schizosaccharomyces pombe* , enabling efficient genome editing (Jacobs et al. 2014; Fernandez and Berro 2016; Rodríguez-López et al. 2017; Zhang et al. 2018; Hayashi and Tanaka 2019). Recently, significant efforts have been made to efficiently clone a guide RNA (gRNA) sequence to a plasmid vector in which gRNA is sandwiched between the *rrk1* promoter/leader sequence and the *rrk1* terminator. Rodríguez-López *et al* . developed a ligation-free method for the PCR-cloning of gRNAs (Rodríguez-López et al. 2017), Jacobs *et al* . developed a cloning-free system using an *in vivo* gap repair process (Jacobs et al. 2014), and Hayashi and Tanaka utilized a type IIS restriction enzyme (BbsI) to create a cloning site for a gRNA, thereby avoiding self-ligation of the backbone plasmid (Hayashi and Tanaka 2019). Despite these advances, it has been suggested that overproduction of Cas9 by the strong *adh1* promoter in these systems can be cytotoxic, thus prompting the need for suitable promoter selection for Cas9 expression (Jacobs et al. 2014; Rodríguez-López et al. 2017; Zhang et al. 2018; Hayashi and Tanaka 2019). Hayashi and Tanaka reported that the expression of Cas9 under the control of the inducible *nmt41* promoter, a weaker version of the *nmt1* promoter, resulted in higher efficiency for gene deletions in comparison to the *nmt1* promoter, emphasizing the significance of promoter activity strength for Cas9 expression in maximizing genome editing efficiency (Hayashi and Tanaka 2019). However, the *nmt* (no message in thiamine) promoters are suppressed in complete medium (because it contains thiamine), necessitating the use of defined minimal media, which can make the entire protocol time-consuming due to the slow cellular growth in the nutrient-poor environments. Although the recently developed *SpEDIT* has addressed the issue by using the *adh15* promoter (a variant of the *adh1* promoter) (Torres-Garcia et al. 2020), a systematic evaluation for the other variants of the *adh1* promoter is still lacking. In the present study, we evaluated whether the weaker derivatives of the *adh1* promoter ( *adh11* , *adh41* , and *adh81* ) are suitable for expression of Cas9 compared to the *adh1* promoter in complete medium (Yokobayashi and Watanabe 2005; Kawashima et al. 2007; Sakuno et al. 2009; Tada et al. 2011; Shichino et al. 2014; Chen et al. 2017), and demonstrated that the *adh11* and *adh41* promoters can support fast and accurate genome editing by the CRISPR/Cas9 system.

In order to evaluate the effect of the *adh1* promoter derivatives, we generated a series of gRNA-Cas9 dual expression vectors (pMS1, pMS11, pMS41, and pMS81) by replacing the *nmt41* promoter in pAH243 (Hayashi and Tanaka 2019) with either of the *adh1* , *adh11* , *adh41* , or *adh81* promoters, respectively (Figure 1A and B). Additionally, we replaced the *ura4* ^+^ selection marker with *hphMX6* , to enable the use of these vectors in both prototrophic and auxotrophic strains. As with the original pAH243, gRNA sequences can be easily cloned into these vectors by using the BbsI restriction sites between the *rrk1* promoter/leader sequence and the *rrk1* terminator. We cloned an *ade6* -gRNA sequence into these expression vectors to generate *ade6* -targeting plasmids: pMS1_ *ade6* -gRNA, pMS11_ *ade6* -gRNA, pMS41_ *ade6* -gRNA, and pMS81_ *ade6* -gRNA. The wild-type 972 *h* ^-^ cells were transformed with these plasmids and plated on adenine-poor YE selective plates containing hygromycin B for 5 days (for pMS1_ *ade6* -gRNA or pMS11_ *ade6* -gRNA transformants) or 3 days (for pMS41_ *ade6* -gRNA or pMS81_ *ade6* -gRNA transformants) at 30 °C. The efficiency of mutagenesis at the *ade6* ^+^ locus was evaluated by the frequency of the appearance of red-colored colonies (Jacobs et al. 2014; Hayashi and Tanaka 2019). While the transformants with the gRNA-empty plasmids did not show red colonies, the transformants with the plasmids carrying the *ade6* -gRNA sequence showed both red and white colonies (Figure 1C). Our results clearly indicated that the number of colonies that appeared after the transformation of the targeting gRNA expression plasmids was significantly smaller than the empty plasmid controls, which is in line with a previous report that suggested that a combination of a targeted gRNA and Cas9 expression threatened cell survival (Jacobs et al. 2014). Transformation by the pMS1_ *ade6* -gRNA plasmid yielded an extremely small number of colonies (i.e., 7), most likely due to the cytotoxic effect of the Cas9 overproduction, and none of these colonies were red. In contrast, intermediate levels of Cas9 expression by the *adh11* or *adh41* promoters resulted in an increase in colony number on the selection plates, with approximately 70–80 % of these colonies being red, indicating efficient mutagenesis (Figure 1C and D). The *adh81* promoter, the weakest among the tested promoters, also supports mutagenesis, albeit with lower efficiency (approximately 50 %) compared to the *adh11* and *adh41* promoters (Figure 1C and D). These results suggest that the *adh11* or *adh41* promoter is suitable for Cas9 expression for the mutagenesis at the *ade6* ^+^ locus.

The efficiency of gene deletion at the *ade6* ^+^ locus was evaluated using pMS11_ *ade6* -gRNA or pMS41_ *ade6* -gRNA. The wild-type cells were co-transformed with these plasmids and a DNA template for gene deletion by homologous recombination repair, then plated on adenine-rich YES selective plates containing hygromycin B and 200 µg/mL adenine. Colonies that appeared on the selective plates at day 5 (for pMS11_ *ade6* -gRNA transformants) or day 3 (for pMS41_ *ade6* -gRNA transformants) were picked up and re-streaked on adenine-poor YE plates. The fractions of red color colonies for pMS11_ *ade6* -gRNA and pMS41_ *ade6* -gRNA were 92 % (59/64) and 97 % (62/64), respectively (Figure 1E). The *ade6* ^+^ open reading frame of red-colored colonies was confirmed by colony PCR to be efficiently deleted in both cases (94 % and 100 % in pMS11_ *ade6* -gRNA and pMS41_ *ade6* -gRNA, respectively) (Figure 1F).

In summary, we demonstrated that weaker versions of the *adh1* promoters can be used for expression of the potentially cytotoxic Cas9 in the fission yeast CRISPR-Cas9 system. Among the tested promoters, the *adh41* promoter emerged as the first choice due to its reasonable success rates in both mutagenesis and gene deletion, and the relatively fast growth of the transformants for the gRNA-Cas9 expression plasmids. However, it cannot be ruled out that the *adh11* or *adh81* promoters could give better results for gene loci other than *ade6* ^+^ or host strains with different genetic backgrounds. In any case, our results highlighted the importance of modulating Cas9 expression levels to optimize genome editing protocols using the CRISPR-Cas9 system. The dual expression plasmids generated in this study are available in the National BioResource Project (see the Data availability section in Methods).

## Methods

Yeast strain and media

The *Schizosaccharomyces pombe* strain 972 *h* ^-^ was used as the parental strain for all transformations. Yeast cells were grown at 30 °C at 140 rpm in YE medium (0.5 % yeast extract, 2 % glucose) and YES medium (YE with supplements, 100 µg/mL uracil, 200 µg/mL adenine, 200 µg/mL leucine, and 200 µg/mL histidine) as previously described (Moreno et al. 1991). YE and YES with or without 100 µg/mL hygromycin B containing 2 % agarose were used for transformation and red-white selection. For solid media, the glucose concentration of YE or YES medium was 3 %.

Construction of plasmids

For the replacement of *ura4* ^+^ with *hphMX6* of the plasmid pAH243, the 1.6-kbp AvrII and SbfI fragment of pFA6a-hphMX6 was ligated into the AvrII and SbfI site of pAH243 (Hayashi and Tanaka 2019), to yield pHN252. To generate *adh1* promoter derivatives, the wild-type promoter sequence (Ch III: 1590564–1591305) was sub-cloned into a cloning vector. Then, mutations within TATA box (5’-TATAAAATA-3’) were introduced by inverse PCR using primers PHN291 (common forward primer) and PHN292 for *adh11* , PHN294 for *adh41* , and PHN295 for *adh81* (lower case sequences of PHN292, PHN294, and PHN295 show mutated TATA box of the *adh1* promoter). Primer sets of PMS1586 and PMS1587, and PMS1588 and PMS1589 were used to separately amplify the *adh1* promoter derivatives sequence by PCR using 972 *h* ^-^ genomic DNA and the templates described above. It should be noted that the original BbsI recognition site within the *adh* promoters was mutated by the PCR procedure (underlined sequences of PMS1587 and PMS1588 show a mutated BbsI recognition site). The 12-kbp DNA fragment except the *nmt41* promoter of pHN252 was amplified by PCR using a primer set, PMS1584 and PMS1585. The 12-kbp backbone and the *adh* promoters’ fragments were ligated by Gibson assembly (New England Biolabs) according to the manufacturer’s instructions, to yield pMS1, pMS11, pMS41, and pMS81 containing *adh1* , *adh11* , *adh41,* and *adh81* promoters, respectively. The sequences of the *adh* promoters, Cas9, the *rrk1* promoter, and the *rrk1* terminator were confirmed by sequencing using primers, PMS1590–PMS1603. The *ade6* gRNA sequence was cloned into the BbsI site of pMS1, pMS11, pMS41, and pMS81 to yield pMS1_ *ade6* -gRNA, pMS11_ *ade6* -gRNA, pMS41_ *ade6* -gRNA, or pMS81_ *ade6* -gRNA, respectively, as previously described (Hayashi and Tanaka 2019). Each *ade6* gRNA sequence was confirmed by sequencing using PMS1511.

Design of a primer set for amplifying homologous recombination template DNA

A primer set, PMS1604 and PMS1605, selected by CRISPR4P (http://bahlerweb.cs.ucl.ac.uk/cgi-bin/crispr4p/webapp.py), was used to generate the homologous recombination template DNA by PCR amplification as previously described (Rodríguez-López et al. 2017).

Yeast transformation

Cells were grown to log phase at 30 °C in 10 mL of YES medium. Cells were collected by centrifugation at 3,000 rpm at room temperature for 2 min, mixed with 1 mL of 0.1 M lithium acetate (LiOAc), and then collected by centrifugation at 10,000 rpm at room temperature for 1 min. Cells were mixed again with 1 mL of 0.1 M LiOAc, and then collected by centrifugation at 10,000 rpm at room temperature for 1 min. After the cells were incubated with 500 µL of 0.1 M LiOAc at 30 °C for 1 hour, 100 µL of an aliquot was mixed with 500 µL of 50 % PEG4000, 60 µL of 1 M LiOAc, 2 µL of 1 mg/mL heat-denatured salmon sperm DNA (Deoxyribonucleic acid, single stranded, from salmon testes; SIGMA), and 1 µg of Cas9 and/or gRNA expressing plasmid (if necessary, 900 pmol of DNA template for deletion by homologous recombination) followed by incubation at 43 °C for 10 min and then left at room temperature for 5 min. Cells were collected by centrifugation at 5,000 rpm at room temperature for 1 min, mixed with 1 mL of YES medium containing 3 % glucose, and then incubated at 30 °C for 4 hours with gentle agitation. Subsequently, cells were collected by centrifugation at 3,000 rpm at room temperature for 2 min, plated on a selective plate (YES or YE with hygromycin B containing 2 % agarose) and then incubated at 30 °C for 3-5 days.

Colony PCR for confirmation of *ade6* ^+^ deletion

A primer set, PMS1606 and PMS1607, selected by CRISPR4P, was used to check the deletion of the *ade6* ^+^ gene as previously described (Rodríguez-López et al. 2017).

Total RNA isolation and Real-Time RT-PCR analysis

Cells transformed with pMS11, pMS41, or pMS81 were cultured in liquid YE medium containing hygromycin B. Total RNA was isolated from logarithmically growing cells using NucleoSpin RNA (TaKaRa) according to the manufacturer’s instructions, and reverse transcribed with Oligo dT primer and random hexamer primers using PrimeScript ^TM^ RT reagent Kit (Perfect Real Time) (TaKaRa) according to the manufacturer’s instructions. Real-time RT-PCR was performed using SYBR Green qPCR Master Mix (TaKaRa) according to the manufacturer’s instructions. For the qPCR reaction, primers PMS1720 and PMS1721 for *Cas9* and PHY144 and PHY145 for the *act1 ^+^* gene were used. To generate standard curves for quantification of expression levels of Cas9 and *act1* ^+^ , a 10-fold dilution series of the pMS11_ *ade6* -gRNA plasmid and the genomic DNA of *S. pombe* strain 972 *h* ^-^ were used as DNA templates, respectively.

Data availability

The plasmids generated in this study (pMS1, pMS11, pMS41, and pMS81) are available from the National BioResource Project (ID numbers FYP5203, FYP5204, FYP5205, and FYP5206, https://yeast.nig.ac.jp/yeast/top.xhtml).

## Reagents

**Table:** 

Primer name	Sequence (5'-3')
PMS1511	AGAAGCTTTGAATAGGTGTTGTAAAGTGTT
PMS1584	TCGAATGGACTATAAGGAC
PMS1585	TGTCTTTTATGGCGATAATTTTC
PMS1586	AATTATCGCCATAAAAGACACCCTACAACAACTAAGAAAATGG
PMS1587	AACGGAGCG CAAGAC CAATGAGACGCGG
PMS1588	CATTG GTCTTG CGCTCCGTTTTTGCTTTG
PMS1589	GGTCCTTATAGTCCATTCGAGAATTCTCTTGCTTAAAGAAAAGCG
PMS1590	GTATTCTGGGCCTCCATGTCCC
PMS1591	CTTATCGTCATCGTCTTTGTAAT
PMS1592	TACAAAGACGATGACGATAAGA
PMS1593	TACCACGAGAAGTACCCCACCA
PMS1594	GAACCTGTCCGACGCCATCCTG
PMS1595	GATTCGCCTGGATGACCAGAAA
PMS1596	GACAGAGAGATGATCGAGGAAC
PMS1597	GAAAACACCCAGCTGCAGAACG
PMS1598	CGCGAGATCAACAACTACCACC
PMS1599	GAAAAGGGCAAGTCCAAGAAAC
PMS1600	TTCAAGTACTTTGACACCACCA
PMS1601	ATCCTAAATCGTATCCAGTACC
PMS1602	AGTGGCACCGAGTCGGTGGTGC
PMS1603	GATCGGACAGTATATTTGATGC
PMS1604	TTGAAAAAAAGATTCGTTTTTTCAACATTTACCATCTCATTAAGCTGAGCTGCCAAGGTATATACATACTTCATCGAATAGCGACCATAGACATAACTGT
PMS1605	TACAATCTAGAATTTCAAAATAAAATATTATTTAAAACAAAAAGCAAGCAAAATCATTTAACAGTTATGTCTATGGTCGCTATTCGATGAAGTATGTATA
PMS1606	ACTGCGCACTAACTCACTACA
PMS1607	CGTCGCAGCACATTATTCGG
PMS1720	ACGTGACCGAGGGAATGAGA
PMS1721	TTTCAGCTGCTTCACGGTCA
PHY144	CCACGCTTGCTTTGAGCTTCA
PHY145	ATCGTCTTGGACTCTGGTGATGG
PHN291	CCATGGGAGGCGTCAAACCGA
PHN292	tataaaGTGGGTGGTGGACAGGTGCC
PHN294	ataaaGTGGGTGGTGGACAGGTGCC
PHN295	atGTGGGTGGTGGACAGGTGCC
